# Association of hyperlipidemia with breast cancer in Bangladeshi women

**DOI:** 10.1186/s12944-021-01480-2

**Published:** 2021-05-22

**Authors:** Fatama Akter Chowdhury, Md Faridul Islam, Mahnaz Tabassum Prova, Mahbuba Khatun, Iffat Sharmin, Kazi Mazharul Islam, Md. Kamrul Hassan, Md. Abdullah Saeed Khan, Mohammed Mostafizur Rahman

**Affiliations:** 1 Department of Plastic Surgery and Burn, Dhaka Medical College Hospital, Dhaka, Bangladesh; 2 Department of Vascular Surgery, National Institute of Cardiovascular Diseases, Dhaka, Bangladesh; 3 Sir Salimullah Medical College Hospital, Dhaka, Bangladesh; 4 Narayanganj General (Victoria) Hospital, Narayanganj, Bangladesh; 5 Abhaynagar Upazila Health Complex, Jashore, Bangladesh; 6 Dhaka Medical College Hospital, Dhaka, Bangladesh; 7 Pi Research Consultancy Center, Dhaka, Bangladesh; 8 Shaheed Syed Nazrul Islam Medical College, Kishoreganj, Bangladesh; 9 Department of Surgery, Bangabandhu Sheikh Mujib Medical University, Dhaka, Bangladesh

**Keywords:** Breast cancer, Malignancy, Lipoprotein, Cholesterol, Hyperlipidemia

## Abstract

**Background:**

The association of circulating lipids with breast cancer is being debated. The objective of this study was to examine the relationship between abnormal plasma lipids and breast cancer risk in Bangladeshi women.

**Methods:**

This was a case-control study designed using a population of 150 women (50 women in each group). The lipid levels of women with breast cancer were compared to the lipid levels of women with benign breast disease (control group 1) and healthy women (control group 2). Study samples were collected from the Department of Surgery, Bangabandhu Sheikh Mujib Medical University, for a period of 1 year. Ethical measures were in compliance with the current Declaration of Helsinki. Statistical analysis was performed with SPSS version 26.

**Results:**

All of the comparison groups shared similar sociodemographic, anthropometric and obstetric characteristics. The incidence of dyslipidemia was significantly higher in breast cancer patients (96%) than in healthy women (84%) and patients with benign breast disease (82%) (*P* < 0.05 for both). The levels of total cholesterol, triglycerides, and low-density lipoprotein (LDL) cholesterol among the breast cancer patient group were significantly higher than those among both benign breast disease patients and healthy women (*P* < 0.05), except for high-density lipoprotein (HDL) cholesterol. Adjusting for other factors, body mass index (BMI) (kg/m^2^) (> 23) [OR 53.65; 95% CI: 5.70–504.73; *P* < 0.001] and total cholesterol (mg/dl) (≥ 200) [OR 16.05; 95% CI: 3.13–82.29; *P* < 0.001] were independently associated with breast cancer.

**Conclusions:**

Total cholesterol and BMI are independent predictors of breast cancer risk among Bangladeshi women.

**Supplementary Information:**

The online version contains supplementary material available at 10.1186/s12944-021-01480-2.

## Background

Breast cancer is the most commonly diagnosed cancer and fifth-leading cause of cancer mortality worldwide. According to the Global Cancer Observatory (GLOBOCON), it accounts for 11.7% (2261419) of new cancer cases and 6.9% (684996) of cancer deaths in 2020, and incident cases are expected to increase by more than 47% by 2040 [1, 2]. Although the developing countries have a low incidence of breast cancer compared to their Western counterparts, it is increasing rapidly [1, 2]. According to a recent estimate, Southeast Asia is burdened with an incidence of 41.2% (age standardized rate [ASR] per 100,000) and 15% mortality (ASR per 100,000) [1]. There are a few reports on the epidemiology and risk factors for breast cancer in Bangladesh. According to a National Institute of Cancer Research and Hospital (NICRH) study, during the period 2005–2010 a total of 5255 breast cancer cases were diagnosed, with an average age of presentation of 42 years [3, 4].

An increase in the number of reported cases and deaths has inspired numerous studies to understand the disease characteristics, causal associations and suitable solutions to mitigate this public health problem. In addition to established risks e.g., higher chronological age [5], genetic influence [6], and being non-Hispanic white [5], several environmental factors [7] play a crucial role beneath in the underlying mechanism. Weight and diet are important environmental factors that influence plasma lipids and lipoproteins, and their role in breast cancer incidence has been a relatively new concept in recent years [8, 9]. The role of cholesterol and its transporters in breast cancer development has been demonstrated in experimental mouse models [10]. The cholesterol metabolite 27-hydroxycholesterol was found to induce proliferation of estrogen receptor-positive breast cancer cells and facilitate metastasis [10, 11]. Activation of different inflammatory pathways by oxidation of lipoproteins and glycation of HDL may have a role in inhibiting apoptosis and augmenting proliferation and migration of cancer cell [12]. Apolipoprotein A-I mimetics and cholesterol-lowering agents are considered potential therapeutic agents to prevent breast cancer by lowering high cholesterol [8]. Thus lipid-lowering agents are giving hope to millions of women who are at risk of breast cancer due to altered lipid levels. Unfortunately, the study results of the influence of dyslipidemia on breast cancer risk are divergent and inconclusive, and no consensus has been made yet. Indeed, one study among humans revealed total cholesterol as a risk factor for breast cancer [13], while findings from several other studies have opposing results [9, 14, 15]. Fewer reports have portrayed an inverse relationship between total cholesterol and the risk of breast cancer [16].

The number of breast cancer cases and related deaths continue to rise in Bangladesh. Hence, understanding the risk factors associated with the disease might aid in the prevention and management of this malignant condition. Therefore, the study was designed to assess the relationship between dyslipidemia and breast cancer among Bangladeshi women.

## Methods

### Study design, study settings and participants

This cross-sectional analytical study was carried out at the Department of Surgery at Bangabandhu Sheikh Mujib Medical University (BSMMU), Shahbag, Dhaka, during the period from July 2018 to September 2019. The inclusion criteria of this study were adult women aged 18 years or above who attended the breast clinic, BSMMU and/or were admitted to the surgery ward of BSMMU. Sample size was determined using the following formula.

$$ n=\frac{{\left({z}_{\alpha }+{z}_{\beta}\right)}^2x\ \left({\sigma}_1^2+{\sigma}_2^2\right)}{{\left({\mu}_1-{\mu}_2\right)}^2} $$

Here, n = estimated sample size, μ_1_ = mean of the quantitative variable in women with breast cancer, μ_2_ = mean of the same quantitative variable in women without breast cancer, σ_1_ = standard deviation of the respective quantitative variable in women with breast cancer, σ_2_ = standard deviation of the respective quantitative variable in women without breast cancer, Z_α_ = Z value of the standard normal deviate at a given level of significance, and Z_β_ = Z value of standard normal deviate at a given power. Taking the corresponding values from a previous study by Owiredu et al. [17] (μ_1_ = 202.00, μ_2_ = 174.50, σ_1_ = 53.60 and σ_2_ = 40.50) and taking a 95% level of significance (Z_α_ = 1.96) and 90% power (Z_β_ = 0.90), the sample size for each group was 48.81. Finally, a total of 50 participants were intended to be recruited. First, a total of 50 women with breast cancer were chosen as cases. Then they were individually matched to two control subjects (1. women who did not develop breast cancer but had benign breast disease, and 2. Women who had no breast disease and were apparently healthy) according to age (within 1 year). Therefore, a total of 150 participants were considered for inclusion. Key exclusion criteria involved patients with a history of other malignancies or patients receiving any kind of lipid lowering agents or any hormonal medications, including oral contraceptive pills. Women who had any diagnosed case of inherited metabolic disease that causes lipid abnormalities such as familial dyslipidemia and congenital defects in lipid metabolism were also excluded from the study.

### Diagnosis of patients, staging and grading of tumors

The presence of a breast mass was confirmed on physical examination by the physician or by mammographic study. A digital mammography device (AMULET Innovality, Fujifilm Corporation, Minato City, Tokyo, Japan) was used for mammographic evaluation of participants using mediolateral oblique and craniocaudal projection. The patients were categorized according to the Breast Imaging Reporting and Data System (BIRADS) final assessment categories- 0: need additional imaging or prior examinations: 1, negative; 2, benign; 3, probably benign; 4, suspicious; 5, highly suggestive of malignancy; and 6, known biopsy proven malignancy. Any lesions were further investigated by core needle biopsy. The College of American Pathologists [18] protocols were used for the histopathological diagnosis of breast cancer. Biopsy-proven confirmed cases were enrolled as “cases”, and biopsy-negative breast mass patients were included as the “benign breast disease groups”. Women who attended the clinic due to suspected breast lesions but were negative for breast cancer both clinically and by mammography were included as controls. Before final inclusion of the controls, a brief history was taken from each control about her past and current disease and medication history. A separate consent form was ensured before core-needle biopsy with precounseling of the potential risk, hazards and benefits of the study. Pathologic staging was based on the American Joint Committee on Cancer 8th Edition [19], and a single experienced pathologist reviewed and determined the characteristics of the primary tumor based on size, axillary nodal status, and resection margin according to immunohistochemical (IHC) staining (see Supplementary Tables 1 and 2 for the staging and tumor guidelines used). Blinding was done to reduce bias of the study. Mammography was performed in collaboration with the Department of Radiology and Imaging, BSMMU.

### Data collection procedure

Following obtainment of written informed consent, data collection was performed by the lead investigator with the aid of a structured questionnaire. The information collected in this study consisted of the following: 1) demographic information as well as personal history, such as menstrual history, obstetric history, personal habits, and family history of the disease; 2) duration of the mass, presenting symptoms, relevant clinical history, current medications and treatment; 3) staging and grading of the tumor for the case; and 4) investigations, such as complete blood count, erythrocyte sedimentation rate (ESR), and lipid profile for all subjects. All collected data were recorded in separate case record forms. The researchers were fully aware of the privacy and anonymity of the participants and followed the guidelines of the Declaration of Helsinki throughout the study.

### BMI assessment and categorization

Participant weight (in kilograms) was measured using a digital bathroom scale (Model: Meb 9370, Miyako Electronics Ltd., Bangladesh) and height (in meters) was measured using a standard measuring tape. Then BMI was calculated using standard formula. The WHO recommended Asian criteria [20] for BMI categorization was used for this study. A BMI ≥23 was considered overweight and/or obese and a BMI between 18.5 and 22.9 was considered normal weight.

### Lipid assay

Analysis of blood samples from cases and their matched controls were done in same batches but in random order. Lipid assays were carried out at the Department of Biochemistry and Molecular Biology, BSMMU. Serum lipid measures were standardized with the Centers for Disease Control (CDC, Atlanta, GA) Lipid Standardization Program. Five milliliters of fasting (at least 12 h) venous blood was collected into Vacutainer® plain tubes, and serum was separated by centrifugation and analyzed on the same day by the enzymatic method in an automated analyzer (Beckman Coulter-AU680) for lipid profiles comprising total cholesterol (TC), triglyceride (TG), LDL and HDL. Dyslipidemia was defined according to the reference levels of the National Cholesterol Education Programme (NCEP) Adult Treatment Panel III (ATP III) guidelines, where hypercholesterolemia was marked as TC > 200 mg/dL or hypertriglyceridemia was marked as TG > 150 mg/dL. The LDL value was categorized as high when it exceeds 130 mg/dl, and HDL was considered low if it was below 40 mg/ dL [21]. Subjects were classified as dyslipidemic when any one of the components of the lipid profile except HDL was beyond the upper limit and in cases where the HDL level was below the lower limit.

### Ethical consideration

The study protocol was approved by the Institutional Review Board (IRB), BSMMU (reference number: BSMMU/2018/11716). Written informed consent was obtained from the participants before inclusion.

### Statistical analyses

Data were statistically analyzed using software SPSS (version 26). Values were expressed as the mean ± standard deviation. Chi-square tests and analysis of variance (ANOVA) were used for statistical comparisons of categorical and continuous data respectively. Post hoc analysis by Bonferroni adjustments was performed where appropriate. Odds ratios (ORs) were derived by multinomial logistic regression, along with 95% confidence intervals (CIs). *P* values less than 0.05 were considered statistically significant.

## Results

A total of 150 women participated in this study, with 50 participants in each group, namely, healthy women, benign breast disease patients, and breast cancer patients. The mean ages of healthy participants, benign breast disease patients and breast cancer patients were 47.8 ± 10.0, 49.6 ± 11.5 and 51.1 ± 9.8 years, respectively. The differences in age among groups were not significant (*P* = 0.282). The majority of participants in all three groups were married, had primary education, were housewives, and came from the middle socioeconomic class. These factors were statistically similar across the three groups. Furthermore, the healthy, benign and malignant participant groups had statistically comparable ages of menarche (12.7 ± 1.0, 12.6 ± 1.1 and 12.4 ± 0.9 years, respectively; *P =* 0.412), ages of menopause (46.0 ± 1.2, 45.9 ± 1.1 and 46.4 ± 1.6 years, respectively; *P =* 0.204) and age of first pregnancy (18.4 ± 5.8, 17.7 ± 6.3 and 19.6 ± 3.3 years, respectively; *P* = 0.184). The average BMI was significantly higher (*P* < 0.001) in breast cancer patients (25.1 ± 1.4 kg/m^2^) than in healthy controls (22.5 ± 1.0 kg/m^2^) and benign breast disease patients (22.7 ± 1.1 kg/m^2^) (Table 1).

**Table 1:** Socio-demographic, anthropometric and obstetric characteristics of participants

Variable	Categories	Control	Benign Breast Disease	Breast cancer	***P***-value
Age (years)		47.8 ± 10.0	49.6 ± 11.5	51.1 ± 9.8	0.282
	≥ 45	33 (66)	39 (78)	39 (78)	0.287
	18–45	17 (34.0)	11 (22.0)	11 (22.0)	
Marital Status	Married	47 (94)	46 (92)	50 (100)	0.143
	Single	3 (6)	4 (8)	0	
Education	No education	9 (18)	8 (16)	10 (20)	0.995
	Primary	14 (28)	16 (32)	14 (28)	
	SSC	8 (16)	9 (18)	7 (14)	
	HSC	7 (14)	6 (12)	5 (10)	
	Graduation and above	12 (24)	11 (22)	14 (28)	
Occupation	Housewife	33 (66)	34 (68)	35 (70)	0.891
	Service holder	8 (16)	8 (16)	9 (18)	
	Teacher	6 (12)	4 (8)	5 (10)	
	Others	3 (6)	4 (8)	1 (2)	
Socio-economic status	Middle class	47 (94)	41 (82)	45 (90)	0.194
	Lower class	3 (6)	9 (18)	5 (10)	
Age menarche (years)		12.7 ± 1.0	12.6 ± 1.1	12.4 ± 0.9	0.412
	< 12	6 (12)	8 (16)	8 (16)	0.808
	≥ 12	44 (88)	42 (84)	42 (84)	
Menopause	Yes	35 (70)	39 (78)	42 (84)	0.245
	No	15 (30)	11 (22)	8 (16)	
Age menopause (years)		46.0 ± 1.2	45.9 ± 1.1	46.4 ± 1.6	0.204
	> 46	14 (40.0)	14 (35.9)	22 (52.4)	0.296
	≤ 46	21 (60.0)	25 (64.1)	20 (47.6)	
Age at first pregnancy (years)		18.40 ± 5.8	17.7 ± 6.3	19.6 ± 3.3	0.184
Number of pregnancies		2.8 ± 1.4	2.8 ± 1.4	3.1 ± 1.3	0.575
BMI (kg/m^2^)		22.5 ± 1.0	22.7 ± 1.1	25.1 ± 1.4*	< 0.001
	Overweight (≥23)	15 (30)	21 (42)	49 (98)	< 0.001
	Normal weight (18.5 to < 23)	35 (70)	29 (58)	1 (2)	
Family history of breast cancer	Present	6 (12)	6 (12)	9 (18)	0.608
	Absent	44 (88)	44 (88)	41 (82)	

Data expressed as n(%) and mean ± SD where appropriate; *SSC* Secondary School Certificate, *HSC* Higher Secondary Certificate, *BMI* Body Mass Index

*P*-value was determined by Chi-square test and ANOVA with post-hoc analysis by Bonferroni adjustments where appropriate

*P-value significant at < 0.05 level in comparison to control and benign breast disease

**Fig. 1:**
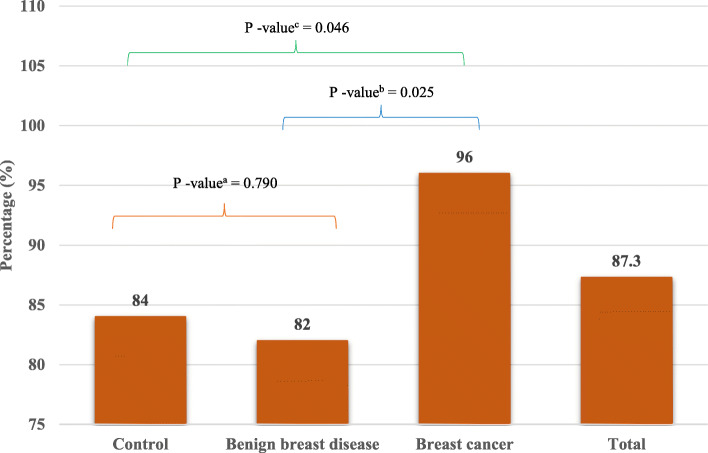
Overall and group specific proportion of dyslipidemia. (Dyslipidemia was defined as having an abnormal value in one or more of the four components of lipid profile. *p*-value as determined by chi-square test between ^a^control and benign breast disease, ^b^benign breast disease and breast cancer, and ^c^control and breast cancer)

Serum lipid profile evaluation of participants showed that TC, LDL and TG were significantly higher among breast cancer patients than among controls and benign breast disease patients (*P* < 0.001). The proportions of hypercholesterolemia among control, benign breast disease and breast cancer patients were 30, 32 and 78%, respectively. Similarly, high LDL was present in 44, 50 and 82% of participants, and high TGs were present in 16, 18 and 52% of participants, respectively. Additionally, the proportions of low HDL among controls, benign breast disease and breast cancer patients were 40, 38 and 62%, respectively. The average values of TC, LDL and TG were significantly higher (*P* < 0.001) in breast cancer patients (224.40 ± 28.09 mg/dl, 142.3 ± 28.43 mg/dl, and 154.92 ± 48.07 mg/dl, respectively) than in controls (187.32 ± 18.93 mg/dl, 126.90 ± 15.16 mg/dl, and 129.28 ± 29.61 mg/dl, respectively) and benign breast disease patients (190.12 ± 15.99 mg/dl, 127.44 ± 14.84 mg/dl and 130.32 ± 30.15 mg/dl, respectively). (Table 2). Overall, 87.3% of participants had dyslipidemia. The proportions of dyslipidemia among controls, benign breast disease patients and breast cancer patients were 84, 82 and 96%, respectively (Fig. 1). The prevalence of dyslipidemia was significantly higher in breast cancer patients than in controls (*P* = 0.046) and benign breast disease patients (*P* = 0.025)*.* However, it was statistically similar between controls and benign breast disease patients (*P* = 0.790). Dyslipidemia was not found to be associated with tumor stage or grade among breast cancer patients (Supplementary Tables 3 and 4). However, a comparison of TC, LDL, HDL, and TG across different stages and grades of tumors showed that TC levels were significantly higher in patients with stage III and IV breast cancer than in those with stage I and stage II breast cancer (*P* < 0.05). Additionally, LDL levels were significantly higher in patients with stage III cancer than in patients with stage II cancer (*P* < 0.05). On the other hand, HDL and TG values were statistically similar across stages of cancer. None of the lipid parameters differed significantly across grades of tumors in breast cancer patients (Supplementary Tables 5 and 6).

**Table 2:** Serum lipid profile of participants

Variable	Categories	Control	Benign Breast Disease	Breast cancer	***P***-value
Total cholesterol (mg/dL)		187.32 ± 18.93	190.12 ± 15.99	220.40 ± 28.09*	< 0.001
	≥ 200	15 (30)	16 (32)	39 (78)	< 0.001
	< 200	35 (70)	34 (68)	11 (22)	
Low density lipoprotein cholesterol (mg/ dL)		126.90 ± 15.16	127.44 ± 14.84	142.30 ± 28.43*	< 0.001
	≥ 130	22 (44)	25 (50)	41 (82)	< 0.001
	< 130	28 (56)	25 (50)	9 (18)	
High-density lipoprotein (mg/ dL)		40.82 ± 6.51	41.14 ± 6.72	39.6 ± 6.98	0.485
	< 40	20 (40)	19 (38)	31 (62)	0.032
	≥ 40	30 (60)	31 (62)	19 (38)	
Triglyceride (mg/dL)		129.28 ± 29.61	130.32 ± 30.15	154.92 ± 48.07*	0.001
	≥ 150	8 (16)	9 (18)	26 (52)	< 0.001
	< 150	42 (84)	41 (82)	24 (48)	

Data expressed as n(%) and mean ± SD where appropriate

*P*-value was determined by Chi-square test and ANOVA with post-hoc analysis by Bonferroni adjustments where appropriate

* *P*-value significant at < 0.05 level in comparison to control and benign breast disease

On univariate multinomial regression analysis, a high BMI (≥23 kg/m^2^), high TC (≥200 mg/dL), high LDL (≥130 mg/dL), low HDL (< 40 mg/dL) and high TG (≥150 mg/dL) were found to be significant predictors of breast cancer. However, none of the factors had a significant association with benign breast disease (Table 3). In multivariate multinomial regression analysis, high BMI (AOR 53.65, 95% CI 5.70–504.73) and high total cholesterol (AOR 16.05, 95% CI 3.13–82.29 mg/dl) were found to be independent risk factors for breast cancer. However, no factors had a significant effect on benign breast disease (Table 4).

**Table 3:** Univariate multinomial regression analysis of risk factors for benign breast disease and breast cancer in relation to controls

Variable	Categories	Benign Breast Disease	***P*** value	Breast cancer	***P-***value
		OR (95% CI)		OR (95% CI)	
Age (years)	≥ 50	1.38 (0.63–3.03)	0.424	1.62 (0.74–3.57)	0.231
	< 50	Ref		Ref	
Menarche age (years)	< 12	1.39 (0.45–4.37)	0.566	1.39 (0.48–4.37)	0.566
	≥ 12	Ref		Ref	
Menopause age (years)	> 46	0.84 (0.33–2.15)	0.716	1.65 (0.67–4.09)	0.280
	≤ 46	Ref		Ref	
BMI (kg/m^2^)	≥ 23	1.69 (0.74–3.86)	0.213	114.33 (14.42–906.25)	< 0.001
	18.5- < 23	Ref		Ref	
Total cholesterol (mg/dL)	≥ 200	1.09 (0.47–2.56)	0.829	8.27 (3.35–20.38)	< 0.001
	< 200	Ref		Ref	
Low density lipoprotein (mg/ dL)	≥ 130	1.27 (0.57–2.79)	0.548	5.79 (2.33–14.44)	< 0.001
	< 130	Ref		Ref	
High-density lipoprotein (mg/ dL)	< 40	0.92 (0.41–2.05)	0.838	2.44 (1.09–5.47)	0.029
	≥ 40	Ref		Ref	
Triglyceride (mg/ dL)	≥ 150	1.15 (0.41–3.28)	0.790	5.69 (2.23–14.53)	< 0.001
	< 150	Ref		Ref	

Data expressed as n(%) and mean ± SD where appropriate; *OR* Odds Ratio

* *P*-value significant at < 0.05 level in comparison to control and benign breast disease

**Table 4:** Multivariate multinomial regression analysis of risk factors for benign breast disease and breast cancer in relation to controls

Variable	Categories	Benign Breast Disease	***P*** value	Breast cancer	***P***-value
		AOR (95% CI)		AOR (95% CI)	
Age (years)	≥ 50	1.10 (0.41–2.95)	0.846	1.88 (0.41–8.58)	0.412
	< 50	Ref		Ref	
Menarche age (years)	< 12	1.29 (0.35–4.69)	0.702	0.43 (0.06–3.26)	0.415
	≥ 12	Ref		Ref	
Menopause age (years)	> 46	0.83 (0.31–2.19)	0.706	1.42 (0.33–6.09)	0.640
	≤ 46	Ref		Ref	
BMI (kg/m^2^)	≥ 23	1.29 (0.49–3.39)	0.594	53.65 (5.70–504.73)	< 0.001
	18.5- < 23	Ref		Ref	
Total cholesterol (mg/ dL)	≥ 200	0.97 (0.29–3.24)	0.967	16.05 (3.13–82.29)	0.001
	< 200	Ref		Ref	
Low density lipoprotein (mg/ dL)	≥ 130	0.96 (0.34–2.67)	0.936	3.015 (0.63–14.51)	0.169
	< 130	Ref		Ref	
High-density lipoprotein (mg/ dL)	< 40	0.81 (0.28–2.34)	0.698	5.01 (0.98–25.52)	0.053
	≥ 40	Ref		Ref	
Triglyceride (mg/ dL)	≥ 150	1.21 (0.35–4.18)	0.758	1.63 (0.32–8.19)	0.552
	< 150	Ref		Ref	

Data expressed as n(%) and mean ± SD where appropriate; *AOR* Adjusted Odds Ratio

*P-value significant at < 0.05 level in comparison to control and benign breast disease

## Discussion

Lipids are essential components of the physiological system of the body. The structural integrity and functional balance of biological membranes depend on their lipid composition. They also play an important role in cellular signaling, working as second messengers and as hormones [22]. Through a yet-to-be-explored mechanism, cholesterol is a risk factor for breast cancer [23]. Additionally, concomitant use of statins with anticancer therapy has been shown to have a protective effect on breast cancer recurrence [24]. Therefore, an investigation of serum lipid profiles in breast cancer might aid in the management of patients. Hence, this study was conducted to explore the association of lipid profiles with breast cancer in patients with breast cancer compared to benign breast disease patients and healthy controls.

An equal number (*n* = 50) of women with breast cancer, benign breast disease, and healthy controls were taken as participants. They had statistically similar sociodemographic and obstetric characteristics. However, a significantly higher BMI was found among breast cancer patients than benign disease patients and healthy controls. This finding is supported by a meta-analysis that evaluated BMI in breast cancer and found that increased weight and obesity have an effect, although minimal, on breast cancer patients [25].

In the present study, 96, 82, and 84% of women with breast cancer, benign breast disease, and normal health, respectively, had dyslipidemia. These proportions are higher than those of a study conducted in Ethiopia [26]. Previously, Sousa-e-Silva et al [27] in Brazil reported a nearly similar proportion of dyslipidemia (90%) among breast cancer patients. However, Li et al [28] in China noted that dyslipidemia was significantly more common among breast cancer patients than among controls, which was also found in this study. One Indian study [29] reported a dyslipidemia prevalence of 21.3% among benign breast disease patients. However, they only explored elevated levels of triglycerides in their research, which indicates that the proportion of dyslipidemia varied from study to study due to the definition used, components of the lipid profile considered, and the ethnic and demographic characteristics of participants.

The current study found that breast cancer patients had significantly higher total cholesterol, LDL, and triglyceride levels than both benign breast disease patients and healthy controls, while the HDL levels remained similar. A concordant finding was reported by researchers in Ghana [17] and India [30]. However, studies conducted in other regions have shown opposing findings. One study in Libya found significantly elevated total cholesterol and HDL levels in breast cancer patients compared with controls [31], while LDL and triglyceride levels remained unchanged in their study. Another study from the Middle East found significantly increased levels of TC and LDL among women with malignant tumors in comparison to the benign and control groups [32]. This was also reported by a Sri Lankan study [33]. A Nepalese study noted an elevation of HDL and a decrease in LDL in breast cancer patients [22]. Ni, Liu, and Gao tried to resolve the inconsistencies and gave a pooled estimate of risks of different lipid profile values for breast cancer [9]. They included fifteen prospective cohort studies in their meta-analysis and found that only serum HDL-C protects against breast cancer among postmenopausal women. However, they also suggested that serum TG might be inversely associated with breast cancer risk rather than directly related. A comparison of the study population with the major findings of these studies are summarized in supplementary Table 7.

A multinomial regression analysis was conducted to determine the relationship between elevated levels of serum lipids and benign breast disease and breast cancer, which was adjusted for biological age, age at menarche, age at menopause, and BMI. Only BMI and total cholesterol were independent predictors of breast cancer, but this was not the case for benign breast disease. In comparison, Laamiri et al. [34] found BMI, TGs, and menopause to be significant predictors of breast cancer risk in multivariate analysis. They also found a protective effect of physical exercise, which was not explored in this study.

A number of contradictory reports exist regarding the association of dyslipidemia with breast cancer risk, which is mainly due to differences in the study population and study area. However, a large number of studies have shown that dyslipidemia in one or more forms exerts an increased risk of breast cancer. Researchers have tried to explore the pathophysiology behind such findings. Nelson, Chang and McDonnell [23] noted that 27-hydroxycholesterol, a cholesterol metabolite, can function as an estrogen, thereby increasing the proliferation of estrogen receptor-positive breast cancer cells. Metabolic syndrome, with dyslipidemia as one of its components, may increase the risk of breast cancer by increasing leptin and decreasing adiponectin levels in blood, as depicted by authors from the USA. These two hormones have been associated with increased breast cancer risk in several studies [35]. Another important link could be the association of hyperlipidemia with increased mammographic breast density [36], which in turn increases the risk of breast cancer [37]. Therefore, dyslipidemia should be taken into consideration while developing strategies for the prevention and treatment of women with breast cancer.

## Limitations of the study

Since this was a single center-based study conducted on a relatively small population carried out within a short period of time, it comes with a set of limitations. The present study did not include long-term follow-ups, and the effect of standard treatment for breast carcinoma on the serum lipid profile was not investigated and therefore omitted. However, the need for these investigations to be included is recognized for future studies.

## Conclusion

The results of the above study showed that there is a significant association between elevated levels of total cholesterol, BMI and breast carcinoma among adult women in Bangladesh. These findings are of importance for the prevention of breast cancer among women with dyslipidemia and overweight or obesity. Additionally, lowering cholesterol and losing weight might aid in the treatment of breast cancer patients.

## Supplementary Information

**Additional file 1: Supplementary Table 1.** Guidelines on histopathological grading of breast cancer. **Supplementary Table 2.** Guidelines on TNM staging of breast cancer. **Supplementary Table 3.** Relationship of dyslipidemia with stages of tumor. **Supplementary Table 4.** Relationship of dyslipidemia with grades of tumor. **Supplementary Table 5.** Serum TC, LDL, HDL and TG across different stages of tumor. **Supplementary Table 6.** Serum TC, LDL, HDL and TG across different grades of tumor. **Supplementary Table 7.** Comparison of findings from studies conducted on the association of lipid profile with breast cancer.

## Data Availability

Data and material would be supplied based on reasonable request.

## References

[1] The ASCO Post Staff. GLOBOCAN. Database provides latest global data on cancer burden. Cancer Deaths. 2021;2020 https://ascopost.com/news/december-2020/globocan-2020-database-provides-latest-global-data-on-cancer-burden-cancer-deaths/.

[2] Heer E, Harper A, Escandor N, Sung H, McCormack V, Fidler-Benaoudia MM (2020). Global burden and trends in premenopausal and postmenopausal breast cancer: a population-based study. Lancet Glob Heal.

[3] Talukder MH, Jabeen S, Islam MJ, Karim N, Shaheen S, Alam AS, et al. Cancer Registry Report National Institue of Cancer Research and Hospital 2005-2007. 2009. https://www.researchgate.net/publication/279851472_Cancer_Registry_Report_2005-2007.

[4] NIRCH. Cancer registry report national institute of cancer research and hospital 2008–2010. 2013; http://nicrh.dghs.gov.bd/about-us.

[5] Breast Cancer Facts & Figures 2019–2020 by American Cancer society, 2020; [cited 2021 Jan 04]. https://www.cancer.org/content/dam/cancer-org/research/cancer-facts-and-statistics/breast-cancer-facts-and-figures/breast-cancer-facts-and-figures-2019-2020.pdf.

[6] Escala-Garcia M, Morra A, Canisius S, Chang-Claude J, Kar S, Zheng W, Bojesen SE, Easton D, Pharoah PDP, Schmidt MK (2020). Breast cancer risk factors and their effects on survival: a Mendelian randomisation study. BMC Med.

[7] Breast Cancer Risk and Environmental Factors. NIH. [cited 2021 Jan 04]. https://www.niehs.nih.gov/health/materials/environmental_factors_and_breast_cancer_risk_508.pdf.

[8] Cedó L, Reddy ST, Mato E, Blanco-Vaca F, Escolà-Gil JC (2019). HDL and LDL: potential new players in breast cancer development. J Clin Med.

[9] Ni H, Liu H, Gao R (2015). Serum lipids and breast cancer risk: a meta-analysis of prospective cohort studies. PLoS One.

[10] Cleary MP, Grande JP, Maihle NJ (2004). Effect of high fat diet on body weight and mammary tumor latency in MMTV-TGF-α mice. Int J Obes.

[11] Llaverias G, Danilo C, Mercier I, Daumer K, Capozza F, Williams TM, Sotgia F, Lisanti MP, Frank PG (2011). Role of cholesterol in the development and progression of breast cancer. Am J Pathol.

[12] Khaidakov M, Mehta JL (2012). Oxidized LDL triggers pro-oncogenic signaling in human breast mammary epithelial cells partly via stimulation of MiR-21. PLoS One.

[13] Kitahara CM, Berrington de González A, Freedman ND, Huxley R, Mok Y, Jee SH (2011). Total cholesterol and cancer risk in a large prospective study in Korea. J Clin Oncol.

[14] Tournberg SA, Holm LE, Carstensen JM (1988). Breast cancer risk in relation to serum cholesterol, serum Beta-lipoprotein, height, weight, and blood pressure. Acta Oncol (Madr).

[15] Martin LJ, Melnichouk O, Huszti E, Connelly PW, Greenberg CV, Minkin S (2015). Serum lipids, lipoproteins, and risk of breast cancer: a nested case-control study using multiple time points. JNCI J Natl Cancer Inst.

[16] His M, Zelek L, Deschasaux M, Pouchieu C, Kesse-Guyot E, Hercberg S, Galan P, Latino-Martel P, Blacher J, Touvier M (2014). Prospective associations between serum biomarkers of lipid metabolism and overall, breast and prostate cancer risk. Eur J Epidemiol.

[17] Owiredu WKBA, Donkor S, Addai BW, Amidu N (2009). Serum lipid profile of breast cancer patients. Pakistan J Biol Sci.

[18] College of American Pathologist. Cancer protocol templates. 2021; [cited 2021 Apr 11]. Available from: https://www.cap.org/protocols-and-guidelines/cancer-reporting-tools/cancer-protocol-templates.

[19] American Joint Committee on Cancer. AJCC cancer staging manual. 8th ed: Springer Nature; 2017. p. 589–625.

[20] WHO expert consultation (2004). Appropriate body-mass index for Asian populations and its implications for policy and intervention strategies. Lancet.

[21] National Cholesterol Education Program (NCEP) Expert Panel on Detection, Evaluation, and Treatment of High Blood Cholesterol in Adults (Adult Treatment Panel III). Third Report of the National Cholesterol Education Program (NCEP) Expert Panel on Detection, Evaluation, and Treatment of High Blood Cholesterol in Adults (Adult Treatment Panel III) final report. Circulation. 2002;106(25):3143-421. https://www.ahajournals.org/doi/10.1161/circ.106.25.3143. 12485966

[22] Pandeya DR, Rajbhandari A, Nepal M, Abdalhabib EK, Bhatta M, Sen MS (2018). Comparative study of serum lipid profiles in nepalese cancer patients attending a tertiary care hospital. Asian Pacific J Cancer Prev..

[23] Nelson ER, Chang C, McDonnell DP (2014). Cholesterol and breast cancer pathophysiology. Trends Endocrinol Metab.

[24] Beckwitt CH, Brufsky A, Oltvai ZN, Wells A (2018). Statin drugs to reduce breast cancer recurrence and mortality. Breast Cancer Res.

[25] Cheraghi Z, Poorolajal J, Hashem T, Esmailnasab N, Doosti IA (2012). Effect of body mass index on breast cancer during premenopausal and postmenopausal periods: a meta-analysis. PLoS One.

[26] Kumie G, Melak T, Baynes HW (2020). The association of serum lipid levels with breast cancer risks among women with breast cancer at felege hiwot comprehensive specialized hospital, Northwest Ethiopia. Breast Cancer Targets Ther.

[27] de Sousa-e-Silva ÉP, Conde DM, Costa-Paiva L, Martinez EZ, Pinto-Neto AM (2014). Risco cardiovascular em mulheres de meia-idade com câncer de mama: Uma comparação entre dois modelos de risco. Rev Bras Ginecol e Obstet.

[28] Li X, Liu ZL, Wu YT, Wu H, Dai W, Arshad B (2018). Status of lipid and lipoprotein in female breast cancer patients at initial diagnosis and during chemotherapy. Lipids Health Dis.

[29] Prabakar M, Prakasam N, Reshma S, Loganathan K, Palani V (2019). A clinical study of serum lipid profile in benign breast disease in a tertiary care hospital. Int Surg J.

[30] Kumar V, Singh A, Sidhu DS, Panag KMDS (2015). A comparitive study to evaluate the role of serum lipid levels in aetiology of carcinoma breast. J Clin Diagnostic Res.

[31] Peela J, Jarari A (2012). The relationship between serum lipids and breast Cancer in Libya. Biochem Anal Biochem.

[32] Abd AA, Nile AK, Al-Wasiti EA, Hussein MM (2019). Assessment of lipid profile parameters in women with benign and malignant breast tumor. Muthanna Med J.

[33] Akalanka HMK, Ekanayake S, Samarasinghe K (2018). Could anthropometric and lipid parameters reflect susceptibility to breast cancer? Comparison of newly diagnosed breast cancer and apparently healthy women. Asian Pacific J Cancer Prev.

[34] Laamiri FZ, Otmani A, Ahid S, Barkat A (2013). Lipid profile among Moroccan overweight women and breast cancer: a case-control study. Int J Gen Med.

[35] Vona-Davis L, Howard-Mcnatt M, Rose DP (2007). Adiposity, type 2 diabetes and the metabolic syndrome in breast cancer. Obes Rev.

[36] Öztürk MA, Keçeci M, Kömoğlu S, Eryilmaz M, Sertbaş YS, Sertbaş M (2018). Hyperlipidemia and mammographic breast density in post-menopausal women. South Clin Istanbul Eurasia.

[37] Nazari SS, Mukherjee P (2018). An overview of mammographic density and its association with breast cancer. Breast Cancer.

