# Supplementary material for: Association of hyperlipidemia with breast cancer in Bangladeshi women
Source: Lipids Health Dis. 2021 May 22;20:52. doi: 10.1186/s12944-021-01480-2 (PMC8141137; doi:10.1186/s12944-021-01480-2)
Supplement: Supplementary file 1 — Additional file 1: Supplementary Table 1. Guidelines on histopathological grading of breast cancer. Supplementary Table 2. Guidelines on TNM staging of breast cancer. Supplementary Table 3. Relationship of dyslipidemia with stages of tumor. Supplementary Table 4. Relationship of dyslipidemia with grades of tumor. Supplementary Table 5. Serum TC, LDL, HDL and TG across different stages of tumor. Supplementary Table 6. Serum TC, LDL, HDL and TG across different grades of tumor. Supplementary Table 7. Comparison of findings from studies conducted on the association of lipid profile with breast cancer. [file 12944_2021_1480_MOESM1_ESM.doc]

**Supplementary Table 1. Guidelines on histopathological grading of breast cancer**

| **Histopathological grading of breast cancer** |
| --- |
| The grade of a tumor indicates what the cells look like and gives an idea of how quickly the cancer may grow and spread. Tumors are graded between 1 and 3. |
| - Grade 1 or well differentiated (score 3, 4, or 5). The cells are slower-growing, and look more like normal breast tissue. |
| - Grade 2 or moderately differentiated (score 6, 7). The cells are growing at a speed of and look like cells somewhere between grades 1 and 3. |
| - Grade 3 or poorly differentiated (score 8, 9). The cancer cells look very different from normal cells and will probably grow and spread faster. |
| (American Joint Committee on Cancer, Cancer Staging Manual, Sixth Edition, 2002, pp. 227-228). |

**Supplementary Table 2. Guidelines on TNM staging of breast cancer**

| **TNM class** | **Criteria** |
| --- | --- |
| T0 | No evidence of primary tumor |
| T1a | Carcinoma in situ |
| T1 | < or = 2 cm |
| T1m1c | Micro invasion 0.1 cm or less |
| T1a | >0.1 to 0.5 cm |
| T1b | >0.5 to 1 cm |
| T1c | >1 to 2 cm |
| T2 | >2 to 5 cm |
| T3 | >5cm |
| T4 | Any size tumor with direct extension to : a) Chest wall or b) skin |
| T4a | Chest wall, not including pectoralis muscle |
| T4b | Skin edema, ulceration, satellite skin nodule |
| T4c | 4a and 4b |
| T4d | Inflammatory carcinoma |
| Nx | Regional lymph nodes cannot be removed |
| N0 | No regional lymph node metastasis |
| N1 | Metastasis to movable ipsilateral axillary lymph nodes |
| N2 | Metastases in ipsilateral axillary lymph nodes fixed of matted (N2a) or met. only in clinically apparent ipsilateral mammary nodes without clinically evident axillary lymph nodes. ( N2b) |
| N3 | Metastases in ipsilateral infra clavicular lymph nodes (N3a) or clinically apparent ipsilateral internal mammary lymph nodes (N3b) or ipsilateral supraclavicular lymph nodes (N3c) |
| MX | Distant metastasis cannot be assessed |
| M0 | No distant metastasis |
| M1 | Distant metastasis |
| **Breast cancer stages** | **Classification criteria based on TNM** |
| Stage 0 | Tis, N0, M0 |
| Stage I | T1, N0, M0 |
| Stage IIA | T0, N1, M0 or  T1, N1, M0 or  T2, N0, M0 |
| Stage IIB | T2, N1, M0 or  T3, N0, M0 |
| Stage IIIA | T0, N2, M0 or  T1, N2, M0 or  T2, N2, M0 or  T3, N1, M0 or  T3, N2, M0 |
| Stage IIIB | T4, N0, M0 or  T4, N1, M0 or  T4, N2, M0 |
| Stage IIIC | any T, N3, M0 |
| Stage IV | any T, any N, M1 |

(American Joint Committee on Cancer, Cancer Staging Manual, Sixth Edition, 2002, pp. 227-228).

**Supplementary Table 3. Relationship of dyslipidemia with stages of tumor**

| Tumor Stage | Dyslipidemia | |  |
| --- | --- | --- | --- |
|  | Present | Absent | p-value |
| Stage I | 9 (100) | 0 | 1.00 |
| Stage II | 29 (93.5) | 2 (6.5) |  |
| Stage III | 7 (100.0) | 0 |  |
| Stage IV | 3 (100.0) | 0 |  |

p-value determined by Fisher’s Exact test; Data was expressed n (%) within rows

**Supplementary Table 4. Relationship of dyslipidemia with grades of tumor**

| Tumor grade | Dyslipidemia | |  |
| --- | --- | --- | --- |
|  | Present | Absent | p-value |
| Grade I | 22 (100.0) | 0 | 0.569 |
| Grade II | 22 (91.7) | 2 (8.3) |  |
| Grade III | 4 (100.0) | 0 |  |

p-value determined by Fisher’s Exact test; Data was expressed n (%) within rows

**Supplementary Table 5. Serum TC, LDL, HDL and TG across different stages of tumor**

| Variables | Tumor Stage | | | | *P-*value |
| --- | --- | --- | --- | --- | --- |
|  | Stage I  (n=9) | Stage II  (n=31) | Stage III  (n=7) | Stage IV  (n=3) |  |
| TC | 208.33 ±7.76 | 212.70 ±26.61 | 262.00 ±9.95^a,b^ | 239.00 ±5.29^a,b,c^ | <0.001 |
| LDL | 142.48 ±5.63 | 134.34 ±28.01 | 166.80 ±21.37^b^ | 166.93 ±15.00 | 0.015 |
| HDL | 37.22 ±5.63 | 40.23 ±7.58 | 37.57 ±2.88 | 45.00 ±9.54 | 0.304 |
| TG | 158.89 ±51.44 | 157.61 ±49.10 | 145.14 ±42.14 | 138.00 ±60.10 | 0.853 |

*P*-value determined by ANOVA with post-hoc tests by Bonferroni and Games-Howell where appropriate. *P*-value significant at <0.05 in comparison to ^a^Stage I, ^b^Stage II, and ^c^Stage III.

**Supplementary Table 6. Serum TC, LDL, HDL and TG across different grades of tumor**

| Variables | Tumor Stage | | | *P-*value |
| --- | --- | --- | --- | --- |
|  | Grade I  (n=22) | Grade II  (n=24) | Grade III  (n=4) |  |
| TC | 211.64 ±19.04 | 224.21 ±33.52 | 245.75 ±14.17 | 0.051 |
| LDL | 142.64 ±17.35 | 138.04 ±36.19 | 166.05 ±12.37 | 0.428 |
| HDL | 40.32 ±6.61 | 38.42 ±7.05 | 42.75 ±8.99 | 0.191 |
| TG | 162.68 ±41.12 | 149.95 ±54.27 | 142.00 ±49.72 | 0.581 |

*P*-value was determined by ANOVA

**Supplementary Table 7. Comparison of findings from studies conducted on the association of lipid profile with breast cancer**

| **Study Features** | **Present** | **Kumar *et al.*[30]** | **Kumie *et al.* [26]** | **Owiredu *et al.*[17]** | **de Sousa-e-Silva *et al.* [27]** | **Li *et al.* [28]** |
| --- | --- | --- | --- | --- | --- | --- |
| **Year** | 2020 | 2015 | 2020 | 2009 | 2014 | 2018 |
| **Country** | Bangladesh | India | Ethiopia | Ghana | Brazil | China |
| **Type and number of participants** | 50 BC  50 BBD  50 NC | 100 BC  100 NC | 23 BC  68 BBD  91 NC | 100 BC  100 NC | 100 BC survivors | 1054 BC  2483 NC |
| **Mean age (years)** | BC – 51.1±9.8  BBD – 49.6±11.5  NC – 47.8 ±10.0  *P=*0.282 | BC – 53.27  NC – 44.55  *P=*NA | BC – 37.65±14.34  BBD – 33.34±10.66  NC – 32.51±9.44  *P=*NA | BC – 48.21±13.69  NC– 42.64±13.40  *P=>0.05* | BC – 53.2±6.0 | BC – 49.93±10.44  NC – 50.16±12.07  *P*=0.569 |
| **BMI (kg/m^2^)** | BC – 25.1 ±1.4  BBD – 22.7 ±1.1  NC – 22.5 ±1.0  *P*<0.001 | BC – 23.32±1.55  NC-22.9±1.64  *P=*0.067 | NA | BC-26.40±4.70  NC-24.90±4.80  P<0.05 | BC-27.8±5.7 | BC-23.44±3.16  NC-22.86±3.09  *P=*<0.001 |
| **Total Cholesterol (mg/dl)** | BC-220.40±28.09  BBD-190.12±15.99  NC-187.32±18.93  *P*<0.001 | BC-177.54±43.24  NC-144.27±31.51  *P=*0.016 | BC-174.43±26.02  BBD-164.76±42.36  NC-141.37±33.21  *P*=0.607 | BC-202.00±53.60  NC-174.50±40.50  *P<*0.001 | NA | BC-172.85±35.96  NC-193.74±36.73  *P=*<0.001 |
| **LDL (mg/dl)** | BC-142.30±28.43  BBD-127.44±14.84  NC-126.90±15.16  *P*<0.001 | BC-106.61±26.63  NC-83.87±29.03  *P=<0.001* | BC-98.52±39.81  BBD-89.24±30.29  NC-90.76±38.06  *P=*0.004 | BC-117.70±42.80  NC-99.60±33.30  *P<*0.001 | NA | BC-108.28±31.32  NC-123.36±33.64  *P=*<0.001 |
| **HDL (mg/dl)** | BC-39.6±6.98  BBD-41.14±6.72  NC-40.82±6.51  *P*<0.001 | BC-39.36±5.52  NC-39.14±6.52  *P=*0.797 | BC-38.26±7.44  BBD-44.69±14.48  NC-47.61±9.12  *P=*0.004 | BC-57.50±33.20  NC-56.80±21.20  P>0.05 | NA | BC-55.3±13.92  NC-57.23±12.37  *P=*<0.001 |
| **Triglyceride (mg/dl)** | BC-154.92±48.07  BBD-130.32±30.15  NC-129.28±29.61s  *P*<0.001 | BC-130.91±39.31  NC-112.08±40.85  *P*=0.001 | BC-211.7±82.92  BBD-170.1±59.02  NC-168.67±41.62  *P=*<0.001 | BC-115.90±68.60  NC-99.60±47.20  *P*<0.05 | NA | BC-112.5±90.35  NC-117.8±76.17  *P=*<0.05 |
| **Overall Dyslipidemia*** | BC-96%  BBD-82%  NC-84%  *P=*0.046 (BC *v* NC)  *P=*0.025 (BC *v* BBD)  *P=*0.790 (BBD *v* NC) | NA | BC-91.3%  BBD-83.82%  NC-64.83%  *P=*NA | NA | BC-90% | BC-42.98%  NC-58.28%  *P=*<0.001 |

**BC: Breast cancer patients; BBD: Benign breast disease patients; NC: Normal or health controls; LDL: Low density lipoprotein; HDL: High density lipoprotein; NA: Not available**

***Dyslipidemia means increased in one or more of total cholesterol, LDL and TG and/or decrease in HDL levels in blood.**
